# Satisfaction and quality of life in women who undergo breast surgery: A qualitative study

**DOI:** 10.1186/1472-6874-9-11

**Published:** 2009-05-01

**Authors:** Anne F Klassen, Andrea L Pusic, Amie Scott, Jennifer Klok, Stefan J Cano

**Affiliations:** 1 Department of Pediatrics, McMaster University, 3A 1200 Main Street W, Hamilton, ON, L8N 3Z5, Canada; 2 Memorial Sloan-Kettering Cancer Center, 1275 York Ave, New York, NY, 10065, USA; 3 University of British Columbia, Vancouver, BC, Canada; 4 Neurological Outcomes Measures Unit, Institute of Neurology, University College London, London, Queen Square, London, WC1N 3BG, England, UK

## Abstract

**Background:**

In cosmetic and reconstructive breast surgery, measurement of patient-reported outcomes has become increasingly important to research efforts and clinical care. We aimed to describe how breast conditions and breast surgery impact on patient satisfaction and quality of life.

**Methods:**

We conducted qualitative, in-depth interviews with 48 women who had undergone either breast reduction (n = 15), breast augmentation (n = 12), or breast reconstruction (n = 21) surgery in order to begin to build a theoretical understanding of patient satisfaction and quality of life in breast surgery patients. Interviews were audio-taped, transcribed verbatim and analyzed thematically.

**Results:**

The patient interviews revealed that breast conditions and breast surgery impact women in the following six main areas: satisfaction with breasts; satisfaction with overall outcome; psychosocial well-being; sexual well-being; physical well-being; and satisfaction with the process of care. We used these six themes to form the basis of a conceptual framework of patient satisfaction and quality of life in women who undergo breast surgery.

**Conclusion:**

Our conceptual framework establishes the main issues of concern for breast surgery patients. This new framework can be used to help develop local guidelines for future clinical assessment, management and measurement, establish the validity of the current management strategies, and develop evidence-based guidance for the development of new patient reported outcome measures for future outcomes research.

## Background

In the United States, over 500,000 women undergo breast surgery procedures each year [1]. Understanding the wide reaching impact of cosmetic and reconstructive breast surgery has thus become increasingly important for clinical research endeavors and surgical quality improvement efforts [2]. Traditional surgical outcomes, centered on morbidity and mortality, remain important but are no longer sufficient on their own. Thus, patient's perceptions of the impact of disease and treatment are increasingly being considered as integral to understanding health outcomes [3-7].

Breast conditions and their associated surgical interventions have a major impact on quality of life. In fact, in specialties such as breast surgery, it has been suggested that "quality of life must be the major if not the only end point" [4]. Despite this, relatively little is known about the extent to which quality of life is impacted in breast surgery populations. There are a number of reasons for this. First, there is a lack of detailed qualitative research, based on inductive research methods, and a paucity of quantitative research, using valid, reliable, and responsive instruments to measure patient-reported outcomes in cosmetic and reconstructive breast surgery [8]. Second, few researchers have tried to understand exactly what having breast conditions means to women, and what impact surgery then has on these perceptions. Third, breast conditions are varied and are associated with complex symptomologies spanning the continuum of impact from physical functioning through to social interaction. As such, women with different conditions may experience the impact of these conditions differently.

It is clear that a thorough evaluation of the impact of breast conditions and their surgical treatment is required. Therefore, in this study we have adopted a qualitative approach [5,9] that involves in-depth interviews with women who had undergone breast surgery (i.e., breast reconstruction, breast reduction, breast augmentation) in order to collect data about their personal experience of breast surgery. This descriptive data was used to develop a theoretical understanding of patient satisfaction and quality of life in breast surgery patients. In particular, we have used detailed analysis [10] to compare and contrast the experiences of these women in order to develop a conceptual framework [11-13] with the view to improving our understanding of the impact of breast conditions and their surgical interventions.

## Methods

### Participants

The sample was recruited from the patients of four plastic surgeons practicing in Vancouver, Canada. These surgeons identified a pool of 120 women who had undergone three forms of breast surgery (i.e., reconstruction, augmentation, reduction). A letter and consent form was sent to each woman from their plastic surgeon inviting her to participate in an in-depth semi-structured interview. Sixty-two women (51.7%) returned a signed consent. Table 1 shows sample characteristics for 48 women (from the 62) that formed the final study sample.

**Table 1:** Characteristics of the study sample

Characteristics	N	%
*Operation type*		

Reduction Augmentation Reconstruction	15 12 21	31.3 25.0 43.8

*Type of reconstruction*		

Implant	12	57.1
Tram	7	33.3
Unilateral implant and tram	2	9.5

*Timing of reconstruction*		

Delayed	9	42.9
Immediate	11	52.4
Unilateral immediate and delayed	1	4.8

*Time since surgery*		

< 12 months 12 to 24 months > 24 months	8 30 10	16.7 62.5 20.8

*Age* 20 – 29 30 – 39 40 – 49 50 – 75	3 14 13 17	6.4 29.8 27.7 36.2

*Marital Status*		

Married Common-law Divorced Widowed Single	21 4 5 2 16	43.8 8.3 10.4 4.2 33.3

*Ethnicity*		

Caucasian Ethnic minority	42 6	87.5 12.5

*Main Activity*		

Working Homemaker Student Retired Unemployed	33 5 3 3 2	70.2 10.6 6.4 6.4 6.4

We obtained local institutional ethics review board approval for this study. Women were invited by mail to participate in an interview where they could tell the *story *of how their breast condition and subsequent surgery had impacted their life. A reminder letter and replacement consent form was sent to non-respondents approximately three weeks after the first mailing. A one-page topic list, developed from a literature review of breast surgery outcome instruments [8], was developed to guide the interviews (see Table 2). This topic list was revised throughout the course of the study, with the findings from earlier interviews influencing and shaping its content. Interviews were initiated by having the participant discuss the circumstances that led to her decision to have surgery. Participants were thereafter encouraged to tell their story as completely as possible in their own words. The researcher consulted the interview guide and asked questions only as necessary to ensure that all topics had been discussed. Interviews were conducted by a trained interviewer (JK) either in the patients' home or a preferred location. All interviews were audio-taped and transcribed verbatim by a professional transcription service.

**Table 2:** Interview guide

**Pre-operation process**: timing; influence/opinion/perceptions of partner, friends, and/or family; reason for operation; motivation; type of operation chosen; information seeking; Internet; decision-making
**Pre/post operation perceptions**: feelings going into the operation; concerns about complications/surgery process; expectations for recovery process; pre-op expectations for results; immediate feelings after operation

**Post-op symptoms**: pain; itchiness; discomfort; mobility problems; fatigue; complications; capsular contracture; rippling; numbness; swelling; movement of the arm; tightness in abdominal area

**Functional ability and role performance**: work and normal activities; interference in social activities; interference in family function; ability to participate in sports/fitness/activities; change in level of comfort; energy and vitality

**Aesthetic outcome**: size; shape; appearance of scar; symmetry; cleavage; appearance of nipple/areola complex; difference in fit of clothing; change in style of clothing; ability to wear desired clothes and styles; body wholeness/harmony; proportionate; feel to touch; breast-self exams; natural

**Psychological well-being and self-concept**: changes in mood; changes in confidence level; emotional distress resulting from teasing, comments, or stares prior to or after operation; body image issues; feelings clothed and unclothed; self-consciousness; self-esteem; feelings of femininity; cancer worry; closure to emotions surrounding disease; feelings of normalcy

**Relationships with friends and family**: reactions of friends and family; difference in treatment or attitude; marital relationship; family relationships; strain of physical or emotional problems on relationships; avoidance behavior; more or less outgoing; feelings in a social setting; undressing in public places

**Sexual life**: satisfaction with sex life; partner's satisfaction; change in frequency of sex; feelings of sexual attractiveness; degree of sensation in breasts; undressing in front of partner

**Surgical care**: satisfaction with care; satisfaction with information provided; comfort with surgeon; confidence in surgeon; surgical setting; clinic; staff; follow-up care; information about scar healing; massaging

**Expectations**: fulfillment of expectations; willingness to repeat and/or recommend procedure; satisfaction with overall appearance; regrets; outcome better or worse than expected; process better or worse than expected

### Data analysis

Data collection and analysis took place concurrently. The iterative interaction between data collection and analysis is the essence of attaining reliability and validity [14] and makes it possible for researchers to pursue emerging avenues of inquiry in further depth [10]. Each transcript was read carefully in order to gain an overview of the main issues of importance to participants. Transcripts were then examined in detail in order to identify basic patterns and recurrent themes using line-by-line coding to examine, compare and begin to develop conceptual categories. Categories were developed inductively using the constant comparison method [10]. Comparing each item with the rest of the data to create analytical categories and then grouping categories together made it possible to identify key themes [10]. All coding was done by one team member (JK) with the study investigators (AP, AK, SC) meeting regularly to discuss the coding results. Interviews were conducted until no new themes were identified through the data analysis. To enhance the accuracy of the account of our research, after completing data analysis, as a form of member-checking [15], we took our ideas back to research participants for their confirmation, holding two focus groups with a total of six women in each group who had undergone breast surgery. Focus groups were led by a trained facilitator who asked participants to discuss the extent to which the important themes that we had identified through our analysis of the interview transcripts reflected their subjective experience.

## Results

Our interview findings with 48 breast surgery patients indicate that breast surgery procedures can clearly affect a woman in multiple spheres of function and quality of life. The analysis revealed the following six key themes that formed the basis of our conceptual framework of patient satisfaction and quality of life in breast surgery patients:

• Satisfaction with breasts

• Satisfaction with overall outcome

• Psychosocial well-being

• Sexual well-being

• Physical well-being

• Satisfaction with the process of care

### Satisfaction with breasts

This theme relates to women's satisfaction with their breasts. Women in all three surgical groups described satisfaction, or lack thereof, with reference to breast size, shape, symmetry, cleavage, scars, positioning, how natural their breasts look and feel, and how their breasts fit in proportion to the rest of the body. A woman who underwent breast augmentation shared:

I have really nice voluptuous rounded normal-sized perky breasts and I am sooo happy with them. Soo happy.

Comments that expressed some dissatisfaction were sometimes qualified by the recognition that although the outcome wasn't perfect, breast appearance was vastly improved by surgery. A reconstructive patient expressed:

So shape-wise, I mean, you know, it's the best it can be given what we have to work with, let's put it that way, but it's not where I'd like it to be.

Women also talked about how their breasts look in bras and clothes. Women in all three surgical groups described how surgery made it possible for them to wear lower cut or tighter fitting tops, and that they now had much more choice in terms of the type of bras, lingerie and swimsuits they could wear. As several women, each in a different surgical group, described:

Some things are much more fun to put on, and the stuff that I used to wear looks way better–I am sure they looked good before, too–but I just fill in a bit more, look a bit more busty in them (Augmentation).

I can fit into regular-sized clothes now, which is a huge difference (Reduction).

I mean, they're not real breasts, and they never will be, but I can go out in a T shirt or buy clothes and they look much better (Reconstruction).

For breast augmentation and reconstruction patients, issues related to the appearance of their implants were discussed, such as rippling and how hard or soft the implants felt to the touch. Specific to reduction and reconstruction patients were issues to do with nipple appearance. A reconstructive patient shared:

I can wear T shirts and because of the nipples, actually, that has been an amazing thing for me, is that I have nipples that show through the T shirt. It just feels normalizing.

Another woman, who underwent breast reduction, stated:

I have one nipple that is sort of misshapen compared to the other one. They aren't exactly the same.

### Satisfaction with overall outcome

This theme relates to an overall sense of satisfaction with the outcome of surgery that women have after going through the process of breast surgery. Women who were satisfied with their surgery overall, expressed how they felt with comments such as:

If I had to do it again, I would do it again (Augmentation).

There is not one day that goes by that I am not so pleased that I did it. (Augmentation)

The bottom line is I really am glad that I did this (Reconstruction).

It just made me feel like I had my body back again (Reconstruction)

I would highly recommend it to anybody who is thinking about it (Reduction).

I am very happy and I don't have any regrets about having the surgery, no matter what. (Reduction)

### Psychosocial well-being

This theme relates to the way that women described the effects of breast surgery on their psychosocial well-being. Women in the three groups talked about how, with surgery, they felt better about themselves in many ways. A common theme was to mention feeling less embarrassed, more confident in a social setting and about their body, and more self-assured.

A breast augmentation patient expressed:

For me it's a confidence thing, to walk into a room and the way my clothes fit now, you know, it's just cause I feel like the rest of my body is proportionate, its how I look in my gym clothes...overall, it has been really, really good...I just feel so much more confident, my self esteem and everything.

A woman who underwent breast reconstruction surgery had the following to say with respect to her outcome:

I have greater self-esteem having been through all this and again because I could come through it and go through the big surgery and come out whole with two breasts...I think that has helped a great deal.

Thirdly, a breast reduction patient shared:

I'm not so embarrassed or trying to hide all the time. So in that way it's better.

Women also talked about feeling more attractive, feminine, good about themselves and normal or like other women. Breast surgery was also seen as a way to bring the body in line with what was perceived to be the "norm" for a woman's body. A number of women who underwent breast reduction, for instance, talked about feeling deformed, or not like other women before surgery. However, with surgery, as one woman expressed:

I feel like a normal person instead of like a freak.

Another breast reduction patient expressed how she felt almost too feminine because of the size of her breasts and how people treated her because of her large breasts. She described feeling:

...almost too feminine when I had big breasts, and that's all people really saw me as.

Women who had undergone reconstruction surgery for breast cancer often expressed how reconstruction was a way to get back what was lost and to move on from the cancer experience. As one woman described:

I think once I had this surgery...it was just closure. It's really like that part of my life didn't happen. It's not denial. I mean I still have to be vigilant and everything its just I got my life back, I really did.

Finally, a breast augmentation summed up her experience as follows:

My confidence level, my self esteem, my self respect, my self worth, everything...it has affected everything. I am just so much more solid, grounded. I feel like I am a whole woman now.

### Sexual well-being

This theme deals with the way that a woman's breast condition and surgery impacts on her sexual life. Negative feelings about ones breasts may interfere with how sexually attractive a woman feels as well as with her sexual functioning and sexual pleasure. With surgery, many women commented that they felt more sexually attractive both when they were clothed and unclothed, more confident sexually, and more satisfied with their sex life. As one breast reduction patient said:

Yes it's better because when they were larger I didn't feel sexy.

And a breast augmentation patient said the following:

What I find now is that I am sensual, which I didn't feel before.

Following surgery, some women expressed concern about changes in their nipple sensation and how this affected sexual pleasure. For instance, one woman shared:

I do really miss my real nipples, because they were really an important part of my sexuality. They are an essential part, and they are something I enjoy.

### Physical well-being

This theme mainly relates to issues surrounding chest and upper body symptoms and how these impact on physical function and participation in activities before and after breast surgery. This theme was discussed in much greater detail by breast reduction and reconstruction patients than augmentation patients.

Reconstruction and reduction patients described a range of chest and upper body symptoms such as arm, shoulder, neck, back and breast pain, as well as tenderness, pulling, discomfort. They also discussed ways in which their breast conditions caused activity limitations, such as difficulty lifting or moving their arms and difficulty doing vigorous activities such as running, playing sports, or exercising, as well as doing everyday household chores. A patient who underwent breast reduction stated:

Putting things into the dishwasher and taking them out has become a totally different experience for me.

Preoperatively, women in the breast reduction group described having painful gouges or grooves in their shoulders from their bra straps, rashes under their breasts, and difficulty sleeping due to breast discomfort. Women in this group were often motivated for surgery due to these physical symptoms, as well as for activity limitations they experienced due to the size of their breasts. A breast reduction patient shared:

Before, I didn't want to run anywhere. Even across the street if something happened I would not run. It was painful and embarrassing.

For women who had reconstructive surgery, pain and activity limitations were often reported and tended to be related to the type of reconstruction and extent of surgery. For example, a woman who underwent Transverse Rectus Myocutaneous Flap (TRAM) surgery described experiencing abdomen weakness. She expressed:

There is sort of a bit of a discomfort there, and I don't feel that I have a lot of strength in my abdomen...the way I used to. So I am pretty cautious about what I am doing exercise-wise.

Another breast reconstruction patient described:

This implant feels as if it is low and I get rib pain.

### Satisfaction with the process of care

Patients in our interviews repeatedly reflected on their satisfaction with process of care issues. Satisfaction with the process of care was clearly an important area in patients overall assessment of the surgery and thus formed an important domain in our conceptual framework. This theme was, however, broad and we identified three main subthemes: satisfaction with preoperative information; satisfaction with the care provided by the plastic surgeon; and satisfaction with the office staff and other members of the medical team.

Satisfaction with information was discussed in terms of general issues applicable to all three surgical groups, such as how the surgery was to be done, healing and recovery time, possible complications that might occur, breast appearance, risks, and scarring. Information needs described by women in our sample were surgery-specific (e.g., differences in types and complications associated with implants were relevant to reconstruction and augmentation but not breast reduction patients).

Patients' relationship with their plastic surgeons was an important aspect of process of care. Women talked about the extent to which their surgeon made them feel comfortable, was caring and reassuring, answered all their questions, understood what they wanted, involved them in the decision-making and provided adequate follow-up. The physician-patient relationship was sometimes mentioned as important in terms of giving the patient confidence to go ahead with surgery. As one woman shared:

My doctor was terrific and I trusted her and I had a lot of confidence in her and it didn't seem like there were an awful lot of things to worry about.

But another woman who underwent a reconstruction felt quite differently:

I had these fears and I just did not feel comfortable discussing them with her.

How women were treated by the medical and office staff was important in terms of satisfaction with the overall experience of care. Women talked about the medical team and the office staff in terms of whether they were professional, treated them with respect, and was kind and friendly. As one patient described:

And once I came home, the home care, I don't know what they called it, but the nurses would come round and they were just excellent. They were all lovely people. They were very positive and very encouraging.

### Formation of the conceptual framework

**Figure 1:**
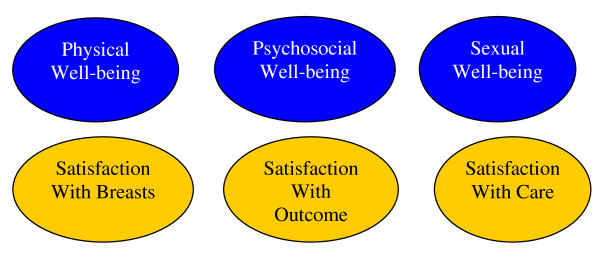
**Conceptual model of patient satisfaction and quality of life in breast surgery patients**.

Relationships between the six main themes described above, which were developed through our detailed coding process, form a coherent and comprehensible conceptual framework of patient satisfaction and quality of life in breast surgery patients. Our conceptual framework is shown in Figure 1.

## Discussion and conclusion

Research that seeks to understand the experiences of any particular patient group needs to employ inductive, qualitative methods. Our goal was to understand issues related to patient satisfaction and quality of life in breast surgery patients and to develop a conceptual framework to better understand the wide reaching impact of breast conditions and the surgical interventions used to treat them.

The patient interviews revealed that breast conditions and breast surgery impact women in six main areas: satisfaction with breasts; satisfaction with overall outcome; psychosocial well-being; sexual well-being; physical well-being; and satisfaction with the process of care. These themes form the basis of a conceptual framework of patient satisfaction and quality of life in women undergoing breast surgery. Patient satisfaction with breast appearance was without doubt the key theme and is a salient factor in determining the success of breast surgery. However, other themes were also identified that related to the broadened notion of quality of life, including concepts such as physical, psychological and sexual well-being. Recognition and examination of these themes confirms findings from existing research showing quality of life benefits following different forms of breast surgery [16-19].

While the six identified themes were common to women in all three groups, the specific issues for operative procedures that preserve or improve breast appearance varied in importance by surgical group. For example, while physical well-being was of only limited importance to breast augmentation patients (only a few reported pain and discomfort post-operatively), it was often the main motivation behind breast reduction surgery (patients reported substantial pain and activity limitations pre-operatively), and was often a problem for women following breast reconstruction. Similarly, while women in the three surgical groups all identified the six themes as being important to them, they expressed themselves differently. As an example, in terms of sexual well-being, an augmentation patient may describe 'feeling sexy' while a reconstruction patient may describe 'feeling normal'.

An important theme within our conceptual framework was that of satisfaction with the process of care. Patients discussed at length the extent to which they had received information about the operation, and their thoughts about their plastic surgeon and his/her medical team and office staff. A clearer understanding of aspects of the processes involved in breast surgery would be a useful addition for quality improvement studies. Using such information could help to determine whether, for example, women who are well informed preoperatively about the surgery (e.g., complications, healing and recovery time, expected results) and feel comfortable with their surgeon, may also report greater postoperative satisfaction and perceive better quality of life.

We are proposing that the six themes identified through patient interviews in this study can be used as the initial building blocks of a conceptual framework to help understand pre- and post-surgical satisfaction and quality of life in breast surgery patients. This new conceptual framework establishes the main issues of concern for breast surgery patients. With further development and input we envisage that this new framework can be used to help develop local guidelines for future clinical assessment, management and measurement, establish the validity of the current management strategies, and develop evidence-based guidance for the development of new patient reported outcome measures for future outcomes research.

We have already taken this work forward by using the conceptual framework to develop a new patient-reported outcome measure. The new measure, which we have named the BREAST-Q^©^, consists of three procedure-specific modules (Augmentation, Reconstruction and Reduction) with each module functioning independently [20]. The items for each module were developed directly from the interview data, and consisting only of items generated by patients who had undergone that procedure. Wherever possible, we maintained the exact wording used by patients for the generation of questionnaire items and ensured that all six themes identified as important to women were captured in each module.

We sought to incorporate patient input at each step in the development of the BREAST-Q^©^. Following the qualitative interviews, women were invited to be part of a focus group where we presented the conceptual framework and our draft questionnaires for their feedback. We also obtained feedback in later phases of our study using one-on-one cognitive debriefing interviews to obtain feedback on our preliminary questionnaires as well as our item-reduced questionnaires. Patient feedback was vital to refining the Breast-Q^©^.

Our team combined our qualitative findings with state-of-the art quantitative psychometric methods that included the use of modern psychometrics (i.e., Rasch analysis) to select the best items from the qualitative interviews for our scales. The use of Rasch analysis makes it possible to select a range of items for each scale that differ in terms of item difficulty such that they "map out" the construct that they propose to measure. The combination of extensive detailed qualitative research and modern psychometric methods make it possible to measure constructs, such as patient satisfaction, in a more clinically meaningful and scientifically robust way than has been done in the past in this patient group.

As described above, the new conceptual framework has value beyond the role it has played in the development of the BREAST-Q^©^. This framework establishes the main issues of concern for breast surgery patients and as such, will be an important resource for healthcare providers and those involved in patient counseling. It may guide the development of patient education materials and facilitate shared-medical decision-making. As well, by conceptualizing patient-perceptions of breast surgery outcomes, it may inform advocacy efforts and future health-services research.

## Competing interests

The authors declare that they have no competing interests.

## Authors' contributions

AK participated in the design of the study, data analysis and interpretation, manuscript writing and final approval of the manuscript. AP conceived and designed the study, participated in collection and assembly of data, data analysis and interpretation, manuscript writing and final approval of the manuscript. AS participated in collection and assembly of data, data analysis and interpretation, manuscript writing and final approval of the manuscript. JK participated in collection and assembly of data, data analysis and interpretation, manuscript writing and final approval of the manuscript. SC participated in the design of the study, collection and assembly of data, data analysis and interpretation, manuscript writing and final approval of the manuscript.

## Pre-publication history

The pre-publication history for this paper can be accessed here:


